# Differential Quantitative Requirements for NPR1 Between Basal Immunity and Systemic Acquired Resistance in *Arabidopsis thaliana*

**DOI:** 10.3389/fpls.2020.570422

**Published:** 2020-09-18

**Authors:** Yezhang Ding, Matthew R. Dommel, Chenggang Wang, Qi Li, Qi Zhao, Xudong Zhang, Shaojun Dai, Zhonglin Mou

**Affiliations:** ^1^ Department of Microbiology and Cell Science, University of Florida, Gainesville, FL, United States; ^2^ Alkali Soil Natural Environmental Science Center, Key Laboratory of Saline-alkali Vegetation Ecology Restoration, Ministry of Education, Northeast Forestry University, Harbin, China

**Keywords:** non-expressor of pathogenesis-related (PR) genes1, systemic acquired resistance, salicylic acid, null mutant, basal immunity, CRISPR mutant, gene induction

## Abstract

Non-expressor of pathogenesis-related (PR) genes1 (NPR1) is a key transcription coactivator of plant basal immunity and systemic acquired resistance (SAR). Two mutant alleles, *npr1-1* and *npr1-3*, have been extensively used for dissecting the role of NPR1 in various signaling pathways. However, it is unknown whether *npr1-1* and *npr1-3* are null mutants. Moreover, the *NPR1* transcript levels are induced two- to threefold upon pathogen infection or salicylic acid (SA) treatment, but the biological relevance of the induction is unclear. Here, we used molecular and biochemical approaches including quantitative PCR, immunoblot analysis, site-directed mutagenesis, and CRISPR/Cas9-mediated gene editing to address these questions. We show that *npr1-3* is a potential null mutant, whereas *npr1-1* is not. We also demonstrated that a truncated npr1 protein longer than the hypothesized npr1-3 protein is not active in SA signaling. Furthermore, we revealed that TGACG-binding (TGA) factors are required for *NPR1* induction, but the reverse TGA box in the 5’UTR of *NPR1* is dispensable for the induction. Finally, we show that full induction of *NPR1* is required for basal immunity, but not for SAR, whereas sufficient basal transcription is essential for full-scale establishment of SAR. Our results indicate that induced transcript accumulation may be differentially required for different functions of a specific gene. Moreover, as *npr1-1* is not a null mutant, we recommend that future research should use *npr1-3* and potential null T-DNA insertion mutants for dissecting NPR1’s function in various physiopathological processes.

## Introduction

Plant systemic acquired resistance (SAR) is a long-lasting immune response against a broad-spectrum of pathogens (Durrant and Dong, 2004). Establishment of SAR largely depends on the signaling molecule salicylic acid (SA) and its receptor non-expressor of pathogenesis-related (PR) genes1 (NPR1) (Delaney et al., 1994; Cao et al., 1997; Wu et al., 2012), also known as non-inducible immunity1 (NIM1) or SA insensitive1 (SAI1) (Ryals et al., 1997; Shah et al., 1997). NPR1 is a coactivator, which controls the expression of a large number of defense genes including *PR* genes through interaction with transcription factors such as the TGACG-binding (TGA) family of bZIP transcription factors (Zhang et al., 1999; Després et al., 2000; Zhou et al., 2000; Subramaniam et al., 2001; Wang et al., 2005).

The functions of NPR1 in SAR, basal immunity, crosstalk between SA and jasmonic acid (JA) signaling, and chemical-mediated defense priming have been well defined using *npr1* mutants (Cao et al., 1994; Ryals et al., 1997; Shah et al., 1997; Zimmerli et al., 2000; Spoel et al., 2003; Leon-Reyes et al., 2009). A large number of *npr1* mutants have been isolated by multiple research groups (Cao et al., 1994; Delaney et al., 1995; Glazebrook et al., 1996; Shah et al., 1997; Canet et al., 2010), among which *npr1-1* and *npr1-3* are the most widely used. The *npr1-1* allele changed a highly conserved histidine (residue 334) in the third ankyrin-repeat to a tyrosine, whereas *npr1-3* introduced a stop codon (residue 400) (Cao et al., 1997). Interestingly, although both *npr1-1* and *npr1-3* are null SAR mutants (Cao et al., 1997), they exhibited significant differences in relation to JA and ethylene (ET) signaling (Glazebrook et al., 2003; Spoel et al., 2003; Leon-Reyes et al., 2009; Canet et al., 2012). These differences were attributed to the existence of a cytosolically localized truncated npr1 (npr1-3) protein that lacks the C-terminal portion with the nuclear localization signal. However, this assumption has never been proven and whether the speculated truncated npr1-3 protein exists or not is still an open question.

In Arabidopsis, the *NPR1* transcripts accumulate constitutively at a low basal level throughout the plant, and the accumulation level can be induced two- to threefold upon pathogen infection or SA treatment (Cao et al., 1997; Ryals et al., 1997). In the 5’ untranslated region (5’UTR) of *NPR1*, there are three W-box (TTGAC) sequences within a 28-bp region from position 103 to 129 upstream of the translation start site (Yu et al., 2001). The third reverse W box overlaps with a TGA-box (TGACG) sequence that is recognized by TGA transcription factors (Thibaud-Nissen et al., 2006). The two adjacent W boxes have been shown to be required for *NPR1* gene induction, but the function of the third W box-TGA box overlapping site is unclear (Yu et al., 2001). Similarly, while W box-binding WRKY transcription factors have been shown to regulate *NPR1* transcription (Yu et al., 2001; Chai et al., 2014), whether TGA factors also participate in the regulation is unknown.

In *npr1* mutants, basal transcript levels of the *npr1* gene are similar to those of the wild type, but SA- and pathogen-mediated induction of the gene is compromised (Ryals et al., 1997; Kinkema et al., 2000; Zhang et al., 2012). These results indicate that NPR1 is required for induction but not for basal transcription of its own gene. Although previous work suggested that basal *NPR1* transcript levels might be sufficient for SAR (van Wees et al., 2000), basal and induced transcript levels of *NPR1* have never been separately evaluated when characterizing NPR1’s function. It remains unknown whether basal NPR1 and induced NPR1 play different functions in some of the signaling processes in which NPR1 is involved.

Here, we show that *npr1-3* is a potential null mutant, whereas *npr1-1* accumulates a low level of mutant protein and should not be considered null. We demonstrated that a truncated npr1 protein longer than the putative npr1-3 protein is not active in SA signaling. Furthermore, we confirmed that NPR1 autoregulates its own gene induction (Ryals et al., 1997; Kinkema et al., 2000; Zhang et al., 2012; Chen et al., 2019), and revealed that TGA factors are required for *NPR1* induction, but the TGA box (the W box-TGA box overlapping site) in the 5’UTR of *NPR1* is dispensable for the induction. Finally, our results show that full induction of *NPR1* is required for basal immunity, but not for SAR, whereas sufficient basal transcription is essential for full-scale establishment of SAR, indicating differential quantitative requirements for NPR1 in these immune responses.

## Materials and Methods

### Plant Materials and Pathogen Infection

The wild types used were the *Arabidopsis thaliana* (L.) Heynh. Columbia (Col-0) and Landsberg *erecta* (L*er*) ecotypes, and the mutant alleles used were *npr1-1*, *npr1-2*, *npr1-3* (Cao et al., 1997), SALK_203386, SALK_204100, SAIL_708_F09, and GT_5_89559 (*npr1-L*, Ding et al., 2015). The transgenic lines *35Spro : NPR1-GFP npr1-2* and *NPR1pro:Myc-NPR1 npr1-3* have been reported previously (Spoel et al., 2009; Zhang et al., 2012). Both transgenes contain the *NPR1* coding region from cDNAs without introns. Arabidopsis seeds were sown on autoclaved soil (Sunshine MVP; Sun Gro Horticulture, Agawam, MA, USA) and cold-treated at 4°C for three days. Plants were germinated and grown at ~23°C under a 16 h light/8 h dark regime.

Inoculation of plants with *Psm* ES4326 was performed by pressure-infiltration with a 1 ml needleless syringe as described previously (Clarke et al., 1998). After inoculation, eight infected leaves were collected for each genotype, treatment, or time point to determine in planta growth of the pathogen. For SAR induction, three lower leaves on each plant were inoculated with the virulent bacterial pathogen *Psm* ES4326 (OD_600_ = 0.002). Two days later, the upper uninfected systemic leaves were either collected for gene expression analysis or challenge-inoculated with *Psm* ES4326 (OD_600_ = 0.001) for resistance test. Eight leaves were collected 3 days after challenge inoculation to examine the pathogen growth.

### Plasmid Construction and Plant Transformation

Site-directed mutagenesis of the TGA box in the 5’UTR of *NPR1* was performed in the previously reported *NPR1pro:Myc-NPR1* construct (Zhang et al., 2012) using a PCR-based Quick-Change site-directed mutagenesis kit (Stratagene, La Jolla, CA). The presence of the expected mutation in the resulting construct was identified by a CAPS marker and verified by DNA sequencing. For creating mutations in the TGA box through gene editing, a nuclease guide sequence (spacer) was introduced into the clustered regularly interspaced short palindromic repeats (CRISPR)/CRISPR-associated protein 9 (CRISPR/Cas9) vector pHSE401 following the published method (Xing et al., 2014). For the *ELP3pro:NPR1* construct, the *ELP3* promoter was amplified from Col-0 genomic DNA, digested with HindIII and BamHI, and cloned into the corresponding sites of the T-DNA binary vector pBI101 (Clontech, Mountain View, CA). The coding region of *NPR1* cDNA was then amplified, digested with BamHI and SacI, and ligated into BamHI/SacI-digested pBI101-ELP3 promoter plasmid. All primers or oligos used in this study were listed in **Table S1**. For plant transformation, the plasmids were introduced into the Agrobacterium strain GV3101(pMP90) by electroporation, and transformation was performed following the floral dip method (Clough and Bent, 1998). Two independent *mNPR1pro:Myc-NPR1* lines, three independent CRISPR mutants, and three independent *ELP3pro:NPR1* lines were characterized in this study

### Chemical Treatment

SA and *β*-aminobutyric acid (BABA) treatments were performed as previously described by Spoel et al. (2009) and Zimmerli et al. (2000), respectively. Briefly, plants were soil-drenched with water solutions containing indicated concentrations of sodium salicylate or BABA. Water treatments were used as the mock controls for both SA and BABA treatments.

### RNA and Protein Analysis

**Figure 1:**
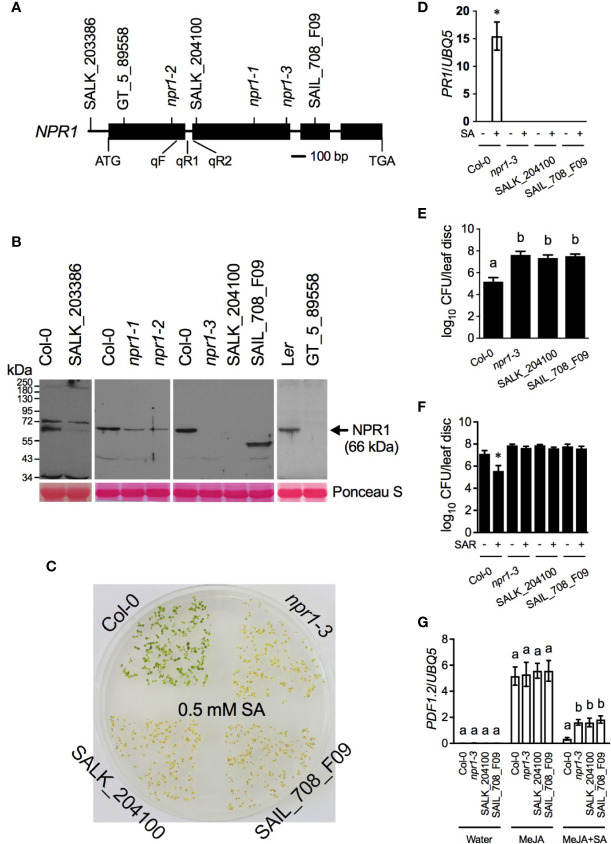
Characterization of multiple *npr1* mutant alleles. **(A)** The T-DNA insertion sites in SALK_203386, GT_5_89558 (*npr1-*L), SALK_204100, and SAIL_708_F09, the positions of the mutations in *npr1-1*, *npr1-2*, and *npr1-3*, as well as the positions of the primers used for qPCR analysis of *NPR1* pre-mRNA (qF + qR1) and mature mRNA (qF + qR2) levels. The precise positions of the T-DNA insertions and mutations are shown in **Table S2**. **(B)** NPR1 protein levels in the wild-type Col-0 and L*er* as well as the indicated *npr1* mutant alleles. Total protein extracted from leaves of 4-week-old soil-grown plants was analyzed by reducing SDS-PAGE and immunoblotting with anti-NPR1 antibody. The arrow indicates the NPR1 band. Ponceau S staining of RuBisCo confirmed equal loading. **(C)** Tolerance of Col-0, *npr1-3*, SALK_204100, and SAIL_708_F09 seedlings to SA toxicity. Seeds were placed on ½ Murashige and Skoog (MS) agar medium containing 0.5 mM SA. After 3 days of stratification, the plate was transferred to a growth chamber and photographed 10 days later. **(D)** SA-induced *PR1* gene expression in Col-0, *npr1-3*, SALK_204100, and SAIL_708-F09. Four-week-old soil-grown plants were treated with soil drenches of 1 mM SA solution (+SA) or water (-SA). Leaf tissues were collected 24 h later. Total RNA was extracted and subjected to qPCR analysis of *PR1* gene expression. Expression was normalized against the constitutively expressed *UBQ5*. Data represent the mean of three independent samples ± standard deviation (SD). The asterisk indicates that *PR1* was significantly induced in Col-0 (*P* < 0.01, Student’s *t*-test). **(E)** Basal resistance of Col-0, *npr1-3*, SALK_204100, and SAIL_708-F09. Four-week-old soil-grown plants were inoculated with a low dose of *Psm* ES4326 (OD_600_ = 0.0001). The *in planta* bacterial titers were determined 3 days postinoculation. Data represent the mean of eight independent samples ± SD. Different letters above the bars indicate significant differences (*P* < 0.05, one-way ANOVA). CFU, colony-forming units. **(F)** Biological induction of SAR in Col-0, *npr1-3*, SALK_204100, and SAIL_708-F09. Three lower leaves on each plant were inoculated with *Psm* ES4326 (OD_600_ = 0.002) (+SAR) or mock-treated with 10 mM MgCl2 (-SAR). Two d later, two upper uninfected/untreated leaves were challenge-inoculated with *Psm* ES4326 (OD_600_ = 0.001). The *in planta* bacterial titers were determined 3 days after challenge inoculation. Data represent the mean of eight independent samples ± SD. The asterisk indicates that *Psm* ES4326 grew significantly less in the SAR-induced plants than in the mock-treated plants (*P* < 0.0001, Student’s *t*-test). **(G)** SA-mediated suppression of MeJA-induced *PDF1.2* gene expression in Col-0, *npr1-3*, SALK_204100, and SAIL_708-F09. Ten-d-old seedlings grown on ½ MS medium were treated with water, 0.1 mM MeJA, or 0.1mM MeJA plus 0.5 mM SA (MeJA+SA). Total RNA was extracted from plant tissues collected 48 h after the treatment and subjected to qPCR analysis of *PDF1.2* expression. Expression was normalized against the constitutively expressed *UBQ5*. Data represent the means of three biological replicates ± SD. Different letters above the bars indicate significant differences (*P* < 0.002, one-way ANOVA). The statistical comparisons were performed among genotypes for each treatment. Experiments in **(B–G)** were repeated three times with similar trend.

Total RNA extraction was carried out as described by Cao et al. (1997). Reverse transcription quantitative PCR (qPCR) was performed as previously described (Defraia et al., 2013) using primers listed in **Table S1**. The *NPR1* mRNA was detected with primers qF and qR1, *NPR1* pre-mRNA was detected with qF and qR2, and *Myc-NPR1* mRNA was detected with the forward primer recognizing a sequence in the *Myc* tag DNA and the reverse primer a sequence in the first exon of the *NPR1* DNA (**Figure 1A** and **Table S1**). Each gene expression analysis experiment was repeated three independent times. In each experiment, three independent biological samples (replicates) were collected at each time point per genotype/treatment and analyzed.

Protein extraction, SDS-PAGE, and immunoblotting were performed as described previously (Mou et al., 2003). The NPR1 and NPR1-GFP proteins were detected using the anti-NPR1 antibody (Ding et al., 2016). Two batches of NPR1 antibodies were used. Both batches detected a specific NPR1 band, and the second batch also detected a non-specific band that is ~6 kDa bigger than NPR1. Ponceau S staining of RuBisCo was used as the loading control. Each immunoblot analysis experiment was repeated at least three independent times, and the result from a representative experiment was presented.

### Statistical Methods

Statistical analyses were performed with the data analysis tools (Student’s *t*-test: Two Samples Assuming Unequal Variances) in Microsoft Excel of Microsoft Office 2004 for Macintosh and the one-way ANOVA in Prism 7 (GraphPad Software, La Jolla, CA).

## Results

### A Truncated npr1 Protein With the N-Terminal 466 Amino Acids Is Inactive in SA Signaling

To address whether truncated npr1 proteins are functional, we tested three T-DNA insertion lines, GT_5_89558 (*npr1-*L) (Ding et al., 2015), SALK_204100, and SAIL_708_F09, which harbor a T-DNA insertion in the first, second, and third exons of the *NPR1* gene, respectively (**Figure 1A**, **Table S2**). These T-DNA insertion lines, together with SALK_203386, which carries a T-DNA insertion in the 5’UTR (**Figure 1A**, **Table S2**), as well as the *npr1-1*, *npr-2*, and *npr1-3* mutants, were subjected to SDS-PAGE immunoblot analysis using the previously reported anti-NPR1 antibody (Ding et al., 2016). As shown in **Figure 1B**, the anti-NPR1 antibody detected a major band at the expected molecular weight of 66 kDa in the wild-type ecotypes, Col-0 and L*er*, but no signal was detected at the expected position in SALK_204100, GT_5_89558, and *npr1-3*. Furthermore, a specific band with a size smaller than that of the wild type was detected in SAIL_708_F09, and a wild-type-size band with significantly reduced intensity was detected in *npr1-1*, *npr1-2*, and SALK_203386. The anti-NPR1 antibody was developed with the N-terminal 465 amino acid residues and the *npr1-3* nonsense mutation is in the codon for residue 400 (Cao et al., 1997; Ding et al., 2016). Although the epitopes recognized by the NPR1 antibody is uncertain, the antibody most likely would detect the truncated npr1-3 protein if it were expressed in the mutant plants. Thus, SALK_204100, GT_5_89558, and *npr1-3* are potential null mutants, SALK_203386 is a knockdown mutant, SAIL_708_F09 is a mutant expressing a truncated npr1 protein, and *npr1-1* as well as *npr1-2* accumulate mutant proteins and are probably not null mutants.

The truncated npr1 protein accumulated in SAIL_708_F09 is 67 amino acids longer than the predicted npr1-3 protein. To test whether this truncated protein is functional in SA signaling, we tested its function in tolerance to SA toxicity, SA-induced *PR1* gene expression, basal resistance, SAR, and crosstalk between SA and JA. As shown in **Figures 1C–G**, SAIL_708_F09 behaved similarly to the potential null mutants *npr1-3* and SALK_204100, indicating that the truncated npr1 protein accumulated in SAIL_708_F09 is not functional in the tested SA responses.

### NPR1 Autoregulates Its Own Gene Induction

**Figure 2:**
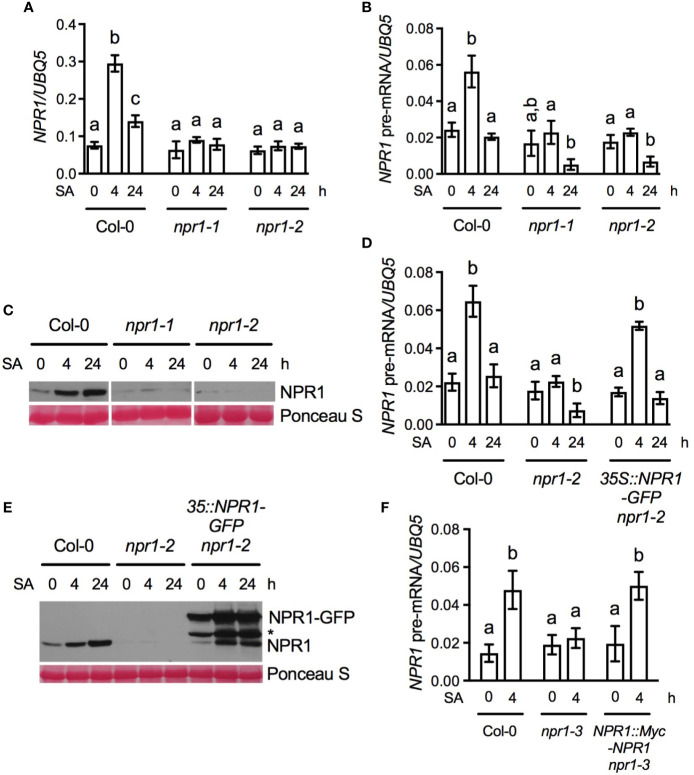
Evidence that NPR1 autoregulates its own gene induction. **(A, B)** SA-induced *NPR1* mature mRNA (A) and pre-mRNA (B) accumulation in Col-0, *npr1-1*, and *npr1-2*. **(C)** SA-induced NPR1 protein accumulation in Col-0, *npr1-1*, and *npr1-2*. **(D)** SA-induced *NPR1* pre-mRNA accumulation in Col-0, *npr1-2*, and *35S:NPR1-GFP npr1-2*. **(E)** SA-induced NPR1 protein accumulation in Col-0, *npr1-2*, and *35S:NPR1-GFP npr1-2*. The asterisk indicates a band with unknown nature. **(F)** SA-induced *NPR1* pre-mRNA accumulation in Col-0, *npr1-3*, and *NPR1:Myc-NPR1 npr1-3*. In **(A, B, D, F)**, 4-week-old soil-grown plants were treated with soil drenches of 1 mM SA solution. Leaf tissues were collected at the indicated time points. Total RNA was extracted and subjected to qPCR analysis. Expression was normalized against the constitutively expressed *UBQ5*. Data represent the mean of three independent samples ± SD. Different letters above the bars indicate significant differences (*P* < 0.05, one-way ANOVA in **(A, B, D)** and Student’s *t*-test in **F**). The statistical comparisons were performed among time points for each genotype. In **(C, E)**, 4-week-old soil-grown plants were treated with soil drenches of 1 mM SA solution. Total protein extracted from leaf tissues collected at the indicated time points was analyzed by reducing SDS-PAGE and immunoblotting with anti-NPR1 antibody. Ponceau S staining of RuBisCo confirmed equal loading. All experiments were repeated three times with similar trend.

To confirm the previous observations that SA- and pathogen-mediated *NPR1* gene induction is compromised in *npr1* mutants (Ryals et al., 1997; Kinkema et al., 2000; Zhang et al., 2012), we treated Col-0, *npr1-1*, and *npr1-2* plants with SA and monitored *NPR1* transcript accumulation. As shown **Figure 2A**, *NPR1* mRNA levels increased approximately threefold 4 h after SA treatment in the Col-0 plants, but did not increase in both *npr1-1* and *npr1-2*. To exclude the possibility that this difference was caused by instability of the *npr1-1* and *npr1-2* mRNA molecules, we monitored *NPR1* pre-mRNA levels by qPCR analysis with the reverse primer in the first intron (**Figure 1A** and **Table S1**). As shown in **Figure 2B**, after SA treatment, *NPR1* pre-mRNA levels were significantly upregulated in Col-0, but not in *npr1-1* and *npr1-2*. Consistent with the observed transcript accumulation, NPR1 protein levels were also dramatically upregulated by SA treatment in Col-0, but not in *npr1-1* and *npr1-2* (**Figure 2C**). Furthermore, the previously reported transgenes *35Spro : NPR1-GFP* and *NPR1pro:Myc-NPR1* restored the SA inducibility of the endogenous *npr1-2* and *npr1-3* genes, respectively (Spoel et al., 2009; Zhang et al., 2012) (**Figures 2D, F**). The npr1-2 protein levels appeared to be also upregulated in the *35Spro : NPR1-GFP* *npr1-2* transgenic plants after SA treatment (**Figure 2E**), though the suspected NPR1 band could be a degradation product of NPR1-GFP. Taken together, these results confirmed that NPR1 is required for its own gene induction (Ryals et al., 1997; Kinkema et al., 2000; Zhang et al., 2012; Chen et al., 2019).

### TGA Transcription Factors Are Required for NPR1 Induction

**Figure 3:**
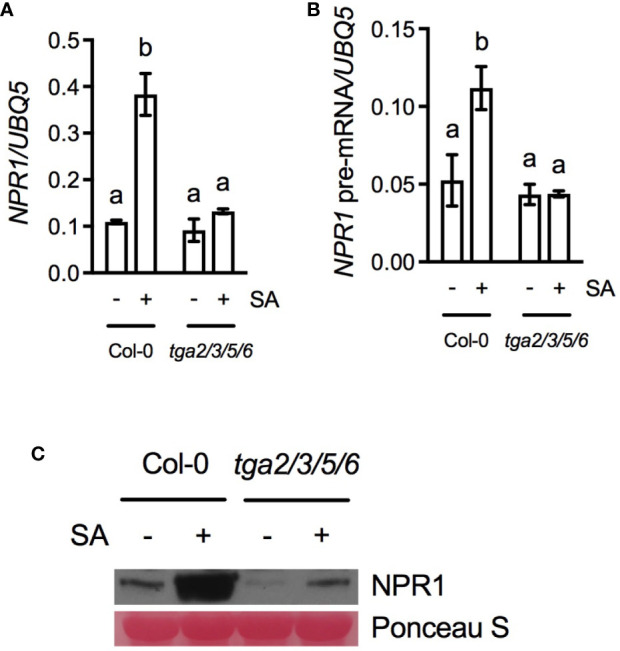
Evidence that TGA factors participate in regulating *NPR1* gene induction. **(A, B)** SA-induced *NPR1* mature mRNA (A) and pre-mRNA **(B)** accumulation in Col-0 and *tga2/3/5/6*. Four-week-old soil-grown plants were treated with soil drenches of 1 mM SA solution (+) or water (-). Leaf tissues were collected 4 h later. Total RNA was extracted and subjected to qPCR analysis. Expression was normalized against the constitutively expressed *UBQ5*. Data represent the mean of three independent samples ± SD. Different letters above the bars indicate significant differences (*P* < 0.03, one-way ANOVA). **(C)** SA-induced NPR1 protein accumulation in Col-0 and *tga2/3/5/6*. Four-week-old soil-grown plants were treated with soil drenches of 1 mM SA solution (+) or water (-). Total protein extracted from leaf tissues collected 24 h later was analyzed by reducing SDS-PAGE and immunoblotting with anti-NPR1 antibody. Ponceau S staining of RuBisCo confirmed equal loading. All experiments were repeated three times with similar trend.

Since NPR1 interacts with a group of TGA transcription factors to regulate defense gene expression (Zhang et al., 1999; Després et al., 2000; Zhou et al., 2000; Subramaniam et al., 2001), we asked whether TGA factors also participate in regulating *NPR1* induction. To this end, we treated Col-0 and the previously reported *tag2/3/5/6* quadruple mutant with SA and monitored *NPR1* transcript and protein accumulation (Kesarwani et al., 2007). As shown **Figures 3A, B**, SA treatment significantly induced both *NPR1* mature mRNA and pre-mRNA accumulation in Col-0, but not in the *tag2/3/5/6* quadruple mutant. Similarly, NPR1 protein levels were dramatically increased in Col-0, but not in the quadruple mutant (**Figure 3C**). These results indicate that TGA factors including TGA2, TGA3, TGA5, and TGA6 are required for SA-mediated *NPR1* induction.

### The TGA Box in the 5’UTR of NPR1 Is Not Required for NPR1 Induction

**Figure 4:**
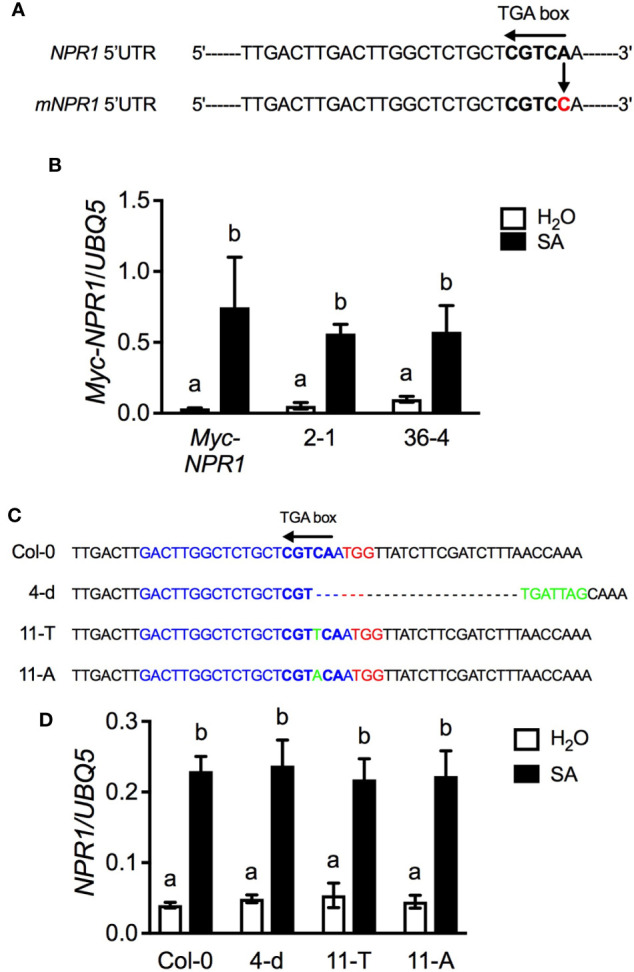
Evidence that the TGA box in the 5’UTR is not required for *NPR1* induction. **(A)** The position of the point mutation made in the TGA box in the 5’UTR of *NPR1*. The mutated nucleotide in the 5’UTR is highlighted in red. **(B)** SA-induced expression of *Myc-NPR1* in *NPR1pro:Myc-NPR1* plants and two independent *mNPR1pro:Myc-NPR1* lines. **(C)** Mutants generated using CRISPR/Cas9-mediated gene editing. The PAM site is highlighted in red, the spacer sequence in blue, and inserted nucleotide in green. “-” indicates deleted nucleotides. **(D)** SA-induced *NPR1* transcript accumulation in Col-0 and three independent CRISPR mutants. In **(B, D)**, 4-week-old soil-grown plants were treated with soil drenches of 1 mM SA solution or water. Leaf tissues were collected 4 h later. Total RNA was extracted and subjected to qPCR analysis. Expression was normalized against the constitutively expressed *UBQ5*. Data represent the mean of three independent samples ± SD. Different letters above the bars in **(B)** and **(D)** indicate significant differences (*P* < 0.05, one-way ANOVA). The experiments were repeated twice **(D)** or three times **(B)** with similar trend.

To test whether the TGA box in the 5’UTR is required for *NPR1* expression, we first made an A-to-C point mutation in the reverse TGA box to change the “CGTCA” sequence to “CGTCC” in the previously reported *NPR1pro:Myc-NPR1* construct (Zhang et al., 2012) (**Figure 4A**), and the resulting construct, *mNPR1pro:Myc-NPR1*, was transformed into the *npr1-3* mutant. Two independent single insertion homozygous *mNPR1pro:Myc-NPR1* lines, 2-1 and 36-4, together with the previously generated *NPR1pro:Myc-NPR1* plants were treated with SA and induction of the transgene transcript accumulation was monitored (Zhang et al., 2012). As shown in **Figure 4B**, SA treatment induced *Myc-NPR1* transcript accumulation to similar levels in all three transgenic lines, indicating that the point mutation introduced into the TGA box in the 5’UTR did not affect the *NPR1* induction.

To confirm the result obtained with the *NPR1pro:Myc-NPR1* transgene, we attempted to create mutations in the TGA box through gene editing. Fortunately, there is a PAM (protospacer adjacent motif) site, TGG, immediately downstream of the reverse TGA box (see Col-0 in **Figure 4C**), which allowed us to use the CRISPR/Cas9 approach to introduce mutations into the TGA box (Xing et al., 2014). As shown in **Figure 4C**, three mutant lines, 4-d, 11-T, and 11-A, were obtained. The TGA box was deleted in line 4-d, and a “T” and an “A” were inserted into the TGA box in lines 11-T and 11-A, respectively. The three CRISPR mutant lines and Col-0 plants were treated with SA and induction of the *NPR1* transcript levels was monitored. As shown in **Figure 4D**, *NPR1* mRNA levels were similarly upregulated in Col-0 and the three CRISPR mutant lines, confirming that the TGA box (the W box-TGA box overlapping site) in the 5’UTR is not required for *NPR1* gene induction.

### Full Induction of NPR1 Is Required for Basal Resistance but Not for BABA-Mediated Priming and Biological Induction of SAR

**Figure 5:**
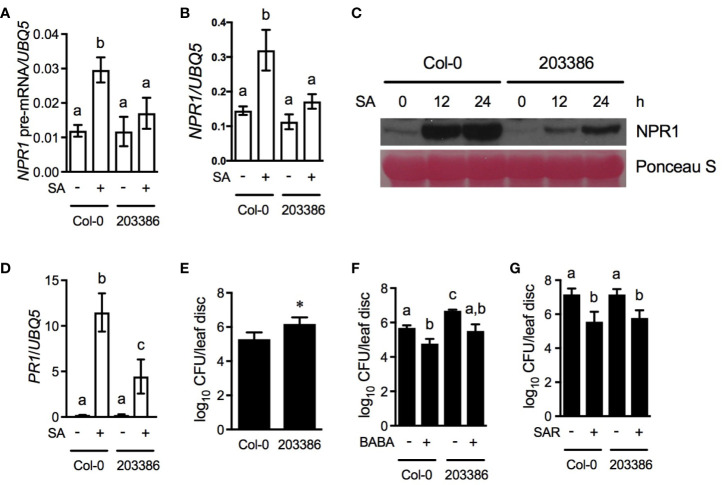
Characterization of a T-DNA insertion line with compromised *NPR1* induction. **(A, B)** SA-induced *NPR1* pre-mRNA (A) and mature mRNA **(B)** accumulation in Col-0 and SALK_203386 (203386). Four-week-old soil-grown plants were treated with soil drenches of 1 mM SA solution (+SA) or water (-SA). Leaf tissues were collected 4 h later. Total RNA was extracted and subjected to qPCR analysis using primer pairs qF + qR1 and qF + qR2 (**Figure 1A** and **Table S1**) for pre-mRNA and mature mRNA, respectively. Expression was normalized against the constitutively expressed *UBQ5*. Data represent the mean of three independent samples ± SD. Different letters above the bars indicate significant differences (*P* < 0.02, one-way ANOVA). **(C)** SA-induced NPR1 protein accumulation in Col-0 and SALK_203386. Four-week-old soil-grown plants were treated with soil drenches of 1 mM SA solution. Total protein extracted from leaf tissues collected at the indicated time points was analyzed by reducing SDS-PAGE and immunoblotting with anti-NPR1 antibody. Ponceau S staining of RuBisCo confirmed equal loading. **(D)** SA-induced *PR1* gene expression in Col-0 and SALK_203386. Total RNA was extracted from leaf tissues collected 24 h after SA treatment and subjected to qPCR analysis. Expression was normalized against the constitutively expressed *UBQ5*. Data represent the mean of three independent samples ± SD. Different letters above the bars indicate significant differences (*P* < 0.001, one-way ANOVA). **(E)** Basal resistance of Col-0 and SALK_203386. Four-week-old soil-grown plants were inoculated with a low dose of *Psm* ES4326 (OD_600_ = 0.0001). The *in planta* bacterial titers were determined 3 d postinoculation. Data represent the mean of eight independent samples ± SD. The asterisk indicates that SALK_203386 is significantly more susceptible than Col-0 to *Psm* ES4326 (*P* < 0.002, Student’s *t*-test). **(F)** BABA-induced resistance in Col-0 and SALK_203386. Four-week-old soil-grown plants were treated with soil drenches of 250 μM of BABA solution (+) or water (-). Two d later, the plants were inoculated with a high dose of *Psm* ES4326 (OD_600_ = 0.001). The *in planta* bacterial titers were determined 3 d postinoculation. Data represent the mean of eight independent samples ± Different letters above the bars indicate significant differences (*P* < 0.05, one-way ANOVA). **(G)** Biological induction of SAR in Col-0 and SALK_203386. Three lower leaves on each plant were inoculated with *Psm* ES4326 (OD_600_ = 0.002) (+SAR) or mock-treated with 10 mM MgCl_2_ (-SAR). Two d later, two upper uninfected/untreated leaves were challenge-inoculated with *Psm* ES4326 (OD_600_ = 0.001). The *in planta* bacterial titers were determined 3 d after challenge inoculation. Data represent the mean of eight independent samples ± SD. Different letters above the bars indicate significant differences (*P* < 0.002, one-way ANOVA). All experiments were repeated three times with similar trend.

To test whether the T-DNA insertion in SALK_203386 affects *NPR1* transcript accumulation, we treated Col-0 and SALK_203386 with SA and monitored *NPR1* transcript levels. As shown in **Figures 5A, B**, induction of both *NPR1* pre-mRNA and mature mRNA levels was significantly reduced in the SALK_203386 plants. Similarly, SA-induced NPR1 protein accumulation was also dramatically inhibited in SALK_203386 (**Figure 5C**). Thus, the inducibility of the *NPR1* gene is largely compromised in SALK_203386. However, SA still induced NPR1 protein accumulation in SALK_203386, which at 12 and 24 h after the treatment reached a level higher than the basal level in the Col-0 plants. The SA-induced elevation of NPR1 protein levels in SALK_203386 may be attributed to the slight, albeit not statistically significant, increase in *NPR1* mRNA levels (**Figure 5B**), and/or SA being able to stabilize the NPR1 protein (Fu et al., 2012; Ding et al., 2016). We found that SA-induced *PR1* expression was also significantly inhibited in SALK_203386 (**Figure 5D**), and that SALK_ 203386 plants were more susceptible than Col-0 to the bacterial pathogen *Psm* ES4326 (**Figure 5E**). On the other hand, treatment with the plant defense-priming compound BABA and biological induction of SAR provided similar levels of resistance to *Psm* ES4326 in the Col-0 and SALK_203386 plants (**Figures 5F, G**). Taken together, these results indicate that the inducibility of *NPR1* is important for basal resistance but not for SAR.

### Sufficient Basal Transcription of NPR1 Is Necessary for Biological Induction of SAR

**Figure 6:**
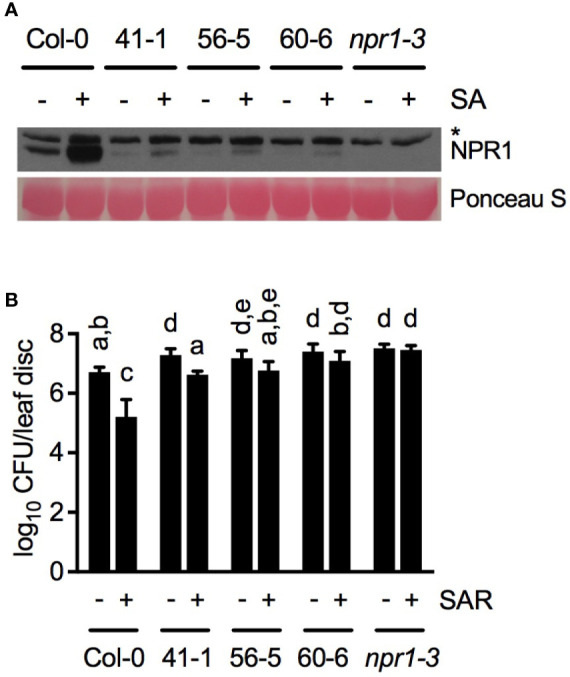
Characterizations of transgenic plants with NPR1 protein levels lower than the wild-type basal level. **(A)** SA-induced NPR1 protein accumulation in Col-0, *npr1-3*, and three independent *ELP3pro:NPR1* transgenic lines. Total protein extracted from leaf tissues collected 24 h after SA (+) or water (-) treatment was analyzed by reducing SDS-PAGE and immunoblotting with anti-NPR1 antibody. The asterisk indicates a non-specific band. **(B)** Biological induction of SAR in Col-0, *npr1-3*, and the three independent *ELP3pro:NPR1* transgenic lines. Three lower leaves on each plant were inoculated with *Psm* ES4326 (OD_600_ = 0.002) (+SAR) or mock-treated with 10 mM MgCl_2_ (-SAR). Two d later, two upper uninfected/untreated leaves were challenge-inoculated with *Psm* ES4326 (OD_600_ = 0.001). The *in planta* bacterial titers were determined 3 d after challenge inoculation. Data represent the mean of eight independent samples ± SD. Different letters above the bars indicate significant differences (*P* < 0.05, one-way ANOVA). The experiments were repeated three times with similar trend.

To evaluate the importance of basal transcription of *NPR1* in SAR, we attempted to generate Arabidopsis plants with NPR1 protein levels lower than the basal level. To this end, we transformed an *ELP3pro:NPR1* construct into the *npr1-3* mutant. We used the *ELP3* promoter, as it confers low-level constitutive gene expression (Defraia et al., 2013). NPR1 protein levels accumulated in three independent transgenic lines, 41-1, 56-5, and 60-6, treated with or without SA were lower than the basal level of NPR1 in Col-0 (**Figure 6A**). We then tested whether the low levels of NPR1 in the transgenic lines are sufficient for SAR induction. As shown in **Figure 6B**, in none of the transgenic lines was SAR induced to the level reached in the Col-0 plants, indicating that sufficient basal transcription of *NPR1* is required for full-scale induction of SAR.

## Discussion

The *npr1-1* and *npr1-3* mutant alleles have been extensively used for dissecting the signaling role of NPR1 in Arabidopsis. Glazebrook et al. (2003) reported that, in response to *Psm* ES4326 infection, the *npr1-3* mutation affected the expression of SA-regulated genes, whereas the *npr1-1* mutation affected not only SA-related genes, but also a much larger group of genes whose expression requires JA and ET signaling. Canet et al. (2012) revealed that methyl JA (MeJA)-induced resistance to the bacterial pathogen *P. syringae* pv. *tomato* (*Pst*) DC3000 was compromised in *npr1-1*, but not in *npr1-3*. Consistently, *npr1-1* was shown to be more susceptible than *npr1-3* to the fungal pathogens *Vericillium longisporum* and *Piriformospora indica* (Johansson et al., 2006; Stein et al., 2008). Furthermore, Leon-Reyes et al. (2009) indicated that SA-mediated suppression of MeJA-induced *PLANT DEFENSIN1.2* (*PDF1.2*) expression was much less affected in *npr1-3* than in *npr1-1*. The differences between *npr1-1* and *npr1-3* have been attributed to a speculated npr1-3 protein (Spoel et al., 2003; Johansson et al., 2006; Stein et al., 2008; Leon-Reyes et al., 2009). Our results indicate that *npr1-3* is a potential null mutant and does not accumulate a truncated form of npr1 (**Figure 1B**). In fact, a truncated NPR1 accumulated in SAIL_708_F09, which is 67 amino acids longer than the hypothesized npr1-3 protein (**Figure 1B**), is not active in multiple SA responses including SA-JA crosstalk (**Figures 1C–G**). Thus, the differences between *npr1-1* and *npr1-3* are likely caused by the npr1-1 protein (**Figure 1B**), which is not active for SA signaling, but may interfere with JA and ET signaling (Canet et al., 2012). Regardless, future research should thus use *npr1-3*, the T-DNA insertion line SALK_204100 (Col-0 background) or GT_5_89558 (L*er* background), for evaluating NPR1’s function in various physiopathological processes.

NPR1 has been shown to autoregulate its own gene transcription (Ryals et al., 1997; Kinkema et al., 2000; Zhang et al., 2012; Chen et al., 2019). We show that the NPR1-interacting TGA transcription factors including TGA2, TGA3, TGA5, and TGA6 are also required for *NPR1* gene induction (**Figure 3**). The *cis*-element characteristic of the TGA factor family is the TGA box that contains the core motif TGACG (Thibaud-Nissen et al., 2006). Intriguingly, mutations of the sole TGA box located in the 5’UTR of *NPR1*, from “TGACG” to “GGACG”, “TGAACG”, “TGTACG”, or “CAACG”, all had no effect on *NPR1* gene induction (**Figure 4**), indicating that the TGA box in the 5’UTR is not required for *NPR1* induction. A potential explanation for this discrepancy could be that TGA factors might regulate *NPR1* induction by acting on an intermediate protein the binds the *NPR1* promoter.

It is well known that pathogen infection induces biosynthesis of SA and expression of SAR-regulating genes including *NPR1* (Durrant and Dong, 2004). van Wees et al. (2000) showed that *NPR1* was not induced in the systemic (upper uninoculated) leaves three days after inoculation of the lower leaves, but the time point might be too late for detecting *NPR1* induction in the systemic leaves (Ding et al., 2016). In this study, we took advantage of the T-DNA insertion line SALK_203386, in which induction of the *NPR1* gene is largely compromised (**Figures 5A, B**), but NPR1 protein can accumulate to a level higher than the basal level in wild type after SA treatment (**Figure 5C**). Results from SALK_203386 revealed that full induction of *NPR1* is required for basal immunity but not for SAR (**Figures 5E, G**), but did not define if an NPR1 level lower than the basal level is sufficient for SAR. To address this question, we created *ELP3pro:NPR1* transgenic lines, in which NPR1 protein levels are lower than the basal level in wild type even after SA treatment (**Figure 6A**). Characterization of the *ELP3pro:NPR1* plants indicated that sufficient basal transcription of *NPR1* is essential not only for basal immunity but also for full-scale establishment of SAR (**Figure 6B**). These results, taken together, suggest differential quantitative requirements for NPR1 between basal immunity and SAR in Arabidopsis. Based on our results, it can be concluded that the NPR1 threshold for full-blown basal immunity is higher than that at which SAR can be fully activated, though it is difficult to accurately determine these thresholds. Interestingly, basal levels of SA have been suggested to be sufficient for SAR induction (Chanda et al., 2011; Gao et al., 2015). It would therefore be possible that, like *NPR1*, basal SA and basal transcription of other SAR-regulating genes are essential for SAR and the induction is necessary for basal immunity. Further investigations are warranted to test this interesting possibility.

## Data Availability Statement

All datasets presented in this study are included in the article/**Supplementary Material**.

## Author Contributions

YD, SD, and ZM designed the experiments. YD, MD, and CW characterized mutants. MD, QL, QZ, and XZ generated and characterized transgenic lines. YD and ZM wrote the manuscript. All authors contributed to the article and approved the submitted version.

## Funding

This work was partially supported by a grant from the University of Florida Research Opportunity Seed Fund (grant no. PRO00018170 awarded to ZM). QZ was supported by a scholarship from the Chinese Scholarship Council.

## Conflict of Interest

The authors declare that the research was conducted in the absence of any commercial or financial relationships that could be construed as a potential conflict of interest.
