# Supplementary material for: Differential Quantitative Requirements for NPR1 Between Basal Immunity and Systemic Acquired Resistance in Arabidopsis thaliana
Source: Front Plant Sci. 2020 Sep 18;11:570422. doi: 10.3389/fpls.2020.570422 (PMC7530841; doi:10.3389/fpls.2020.570422)
Supplement: Supplementary file 1 [file Table_1.docx]

**Supplemental Material:**

**Differential Quantitative Requirements for NPR1 between Basal Immunity and Systemic Acquired Resistance in *Arabidopsis thaliana***

Yezhang Ding, Matthew R. Dommel, Chenggang Wang, Qi Li, Qi Zhao, Xudong Zhang, Shaojun Dai, and Zhonglin Mou

**Table S1.** New primers used in this study

| Target | Forward (5’ to 3’) | Reverse (5’ to 3’) |
| --- | --- | --- |
| *NPR1* pre-mRNA (qF+qR1) | ACGAGAATTGCTGCCACGTG | CCATAGCTTAATGCAGATGGTG |
| *NPR1* mature mRNA (qF+qR2) | ACGAGAATTGCTGCCACGTG | CTACAACGTCCAATAAGTGCC |
| *Myc-NPR1* | TCTGAAGAGGACTTGAATTCC | TGCAGAGCAGATACATCAGG |
| *NPR1* site-directed mutagenesis | GGTTAAAGATCGAAGATAACCATGGACGAGCAGAGCCAAG | CTTGGCTCTGCTCGTCCATGGTTATCTTCGATCTTTAACC |
| Identification of the site-directed mutation (cut by NcoI) | ATTCGAAGCTGTTGGAGAGC | TTCAGAACGAGAAGTTGAAGG |
| CRISPR/Cas9 mutagenesis | ATTGACTTGGCTCTGCTCGTCAA | TTGACGAGCAGAGCCAAGTCAAA |
| The *ELP3* promoter | CCCAAGCTTCTCTACGAAGGCTACGCAC | CGGGATCCGTCACCAAAACCTCAGACC |
| The *NPR1* CDS | CGGGATCCATGGACACCACCATTGATGG | CGAGCTCTCACCGACGACGATGAGAG |

**Table S2.** Positions of the T-DNA insertions and the *npr1* mutations in the plants used in this study

| Mutant | Position* |
| --- | --- |
| SALK_203386 | -144/-145 |
| GT_5_89558 | +119/+120 |
| *npr1-1* | +1079 (C to T; His to Tyr) |
| *npr1-2* | +449 (G to A; Cys to Tyr) |
| *npr1-3* | +1277 (C to T; Gln to Stop) |
| SALK_204100 | +663/+664 |
| SAIL_708_F09 | +1584/+1585 |

*The position is relative to the translation start site (+1) in the genomic DNA.
